# *rac*-*N*-Benzyl­isatincreatinine (unknown solvate)

**DOI:** 10.1107/S1600536813000378

**Published:** 2013-01-26

**Authors:** Narsimha Reddy Penthala, Peter A. Crooks

**Affiliations:** a Department of Pharmaceutical Sciences, College of Pharmacy, University of Arkansas for Medical Sciences, Little Rock, AR 72205, USA

## Abstract

The title compound, C_19_H_18_N_4_O_3_ [systematic name: (*RS*)-1-benzyl-3-hy­droxy-3-(2-imino-3-methyl-5-oxoimidazolidin-4-yl)-2,3-dihydro-1*H*-indol-2-one], was prepared as a racemate (*RR* and *SS*) by the aldol condensation of *N*-benzyl­isatin with creatinine in the presence of sodium acetate in acetic acid. The r.m.s. deviation of the isatin ring system is 0.033 Å. The benzyl group is disordered over two orientations, with refined occupancies of 0.847 (7) and 0.153 (7). The dihedral angles between the isatin ring system and the benzene ring (major disorder component) and the imidazole ring are 82.82 (7) and 51.31 (3)°, respectively, In the crystal, mol­ecules are linked into (001) sheets by N—H⋯O and O—H⋯N hydrogen bonds, which incorporate *R*_2_^2^(9) ring motifs. The crystal was grown from mixed solvents (ethanol, methanol and possibly also ethyl acetate). These solvents are disordered in the crystal and the resulting electron density was found to be uninter­pretable. The solvent contribution to the scattering was removed with the SQUEEZE routine in *PLATON* [Spek (2009 ▶). *Acta Cryst.* D**65**, 148–155]. The formula mass and density do not take account of the solvent.

## Related literature

For details on the development of isatin derivatives as anti­cancer agents, see: Penthala *et al.* (2010*a* ▶,*b* ▶). For similar structures, see: Tang *et al.* (2009 ▶); Penthala *et al.* (2009*a* ▶,*b* ▶).
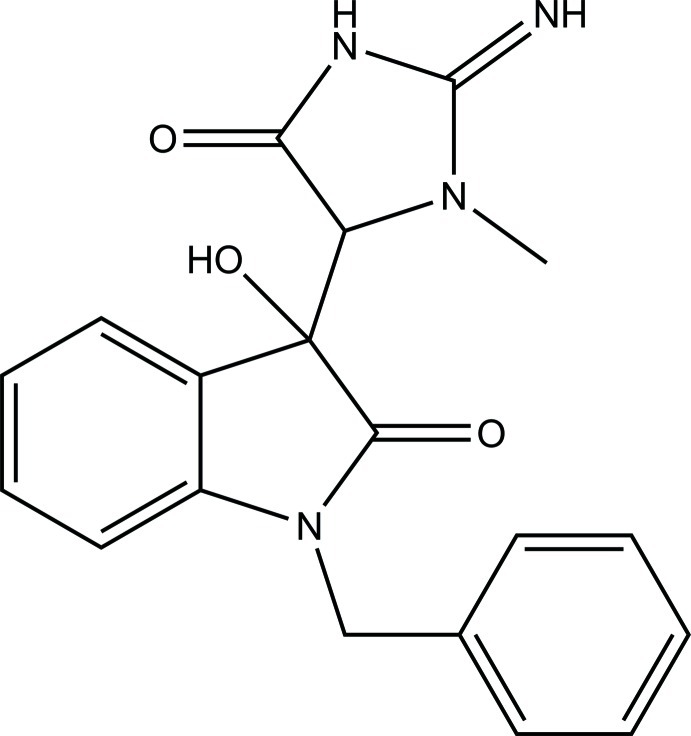


## Experimental

### 

#### Crystal data

C_19_H_18_N_4_O_3_
*M* *_r_* = 350.37
Orthorhombic, 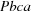

*a* = 13.4466 (2) Å
*b* = 10.6921 (2) Å
*c* = 27.2057 (5) Å
*V* = 3911.43 (12) Å^3^
*Z* = 8
Cu *K*α radiation
μ = 0.68 mm^−1^
*T* = 90 K
0.12 × 0.10 × 0.04 mm

#### Data collection

Bruker X8 Proteum CCD diffractometer
Absorption correction: multi-scan (*SADABS*; Bruker, 2006 ▶) *T*_min_ = 0.911, *T*_max_ = 0.973
55165 measured reflections
3602 independent reflections
3344 reflections with *I* > 2σ(*I*)
*R*_int_ = 0.043

#### Refinement

*R*[*F* ^2^ > 2σ(*F* ^2^)] = 0.038
*wR*(*F* ^2^) = 0.104
*S* = 1.04
3602 reflections
287 parameters
222 restraints
H-atom parameters constrained
Δρ_max_ = 0.28 e Å^−3^
Δρ_min_ = −0.30 e Å^−3^

### 

Data collection: *APEX2* (Bruker, 2006 ▶); cell refinement: *SAINT* (Bruker, 2006 ▶); data reduction: *SAINT*; program(s) used to solve structure: *SHELXS97* (Sheldrick, 2008 ▶); program(s) used to refine structure: *SHELXL97* (Sheldrick, 2008 ▶); molecular graphics: *XP* in *SHELXTL* (Sheldrick, 2008 ▶); software used to prepare material for publication: *SHELX97*.

## Supplementary Material

Crystal structure: contains datablock(s) global, I. DOI: 10.1107/S1600536813000378/hb7013sup1.cif

Structure factors: contains datablock(s) I. DOI: 10.1107/S1600536813000378/hb7013Isup2.hkl

Supplementary material file. DOI: 10.1107/S1600536813000378/hb7013Isup3.cml

Additional supplementary materials:  crystallographic information; 3D view; checkCIF report

## Figures and Tables

**Table 1:** Hydrogen-bond geometry (Å, °)

*D*—H⋯*A*	*D*—H	H⋯*A*	*D*⋯*A*	*D*—H⋯*A*
O9—H9⋯N12^i^	0.84	1.97	2.8065 (13)	175
N13—H13*A*⋯O11^ii^	0.88	2.24	2.9321 (13)	135
N13—H13*B*⋯O1^iii^	0.88	1.97	2.8410 (14)	173

Symmetry codes: (i) 
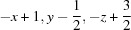
; (ii) 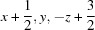; (iii) 
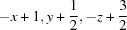
.

**Fig. 1.**
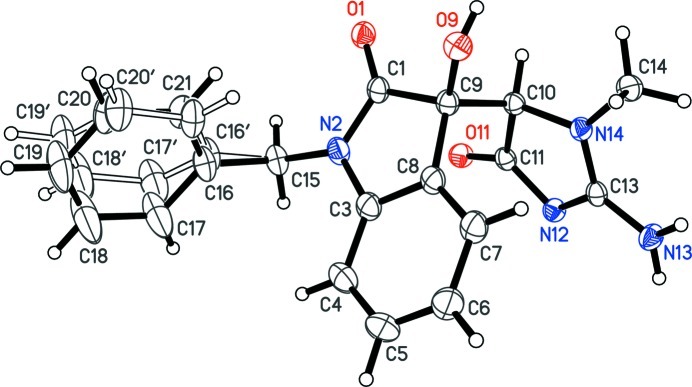
A view of (I). Displacement ellipsoids are drawn at the 50% probability level.

**Fig. 2.**
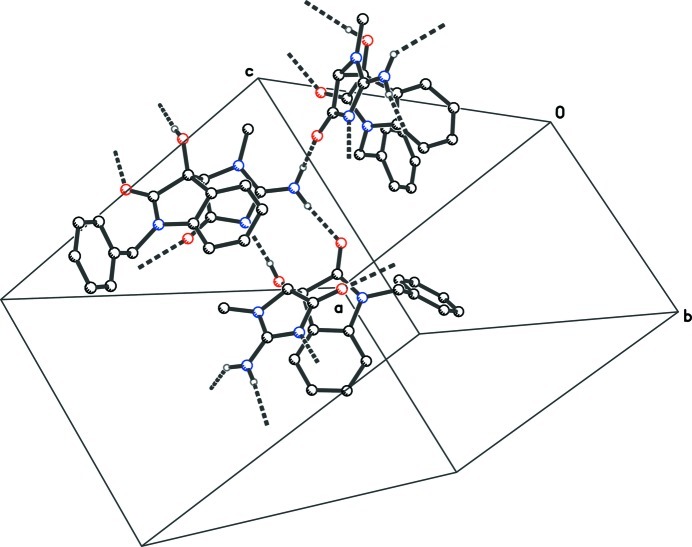
Hydrogen bonding in the crystal structure of (I). Dashed lines represent hydrogen bonds.

## supplementary crystallographic information

### Comment

In continuation of our work on the development of anti-cancer agents (Penthala *et al.*, 2010*a*,*b*), we have synthesized a series of new compounds containing isatin and creatinine moieties to screen for anticancer activity against a panel of 60 human cancer cell lines (Penthala *et al.*, 2010*a*). The title compound was prepared by the aldol condensation of *N*-benzylindol-2,3-dione (*N*-benzylisatin) with 2-amino-1-methyl-1*H*-imidazol-4(5*H*)-one (creatinine) in the presence of sodium acetate in acetic acid. Earlier, we reported on the crystal structure of isatin creatinine analogs containing *N*-methyl and *N*-phenyl substituents (Penthala *et al.*, 2009*a*,*b*). To obtain detailed information on the structural conformations of the molecules for analysis of structure-activity relationships (SAR), we determined the X-ray crystal structure of the title compound (Fig. 1). In the crystal, the benzyl group is disordered over two positions, with refined occupancies of 0.847 (7) and 0.153 (7). The isatin ring is almost planar, with r.m.s deviations from the mean plane = 0.0508 (11) Å, and with bond distances and angles comparable to those reported for other isatin derivatives (Tang *et al.*, 2009). The benzene ring of the benzyl group makes a dihedral angle with the mean plane of the isatin ring of 82.82 (7)°. The title compound was isolated as a racemate (*RR* and *SS*). In the crystal, the molecules are linked into 2-D pleated-sheet networks in the *ab* plane by a series of intermolecular N—H—O and O—H—N hydrogen bonds. Within these sheets, the hydrogen bonds O9—H···N12, N13—H13A···O11 and N13—H13B···O1 create *R*^2^_2_(9) ring motifs.

### Experimental

The title compound was prepared according to a previously reported procedure (Penthala *et al.*, 2009*a*,*b*). Recrystallization from ethanol afforded the title compound as pale yellow plates. Spectroscopic data for *rac*-*N*-benzylisatincreatinine: ^1^H NMR (DMSO-*d_6_*): δ 3.17 (s, 3H, CH_3_), 4.21 (s, 1H, CH), 4.74–4.91 (ABq, *J*= 16.2 Hz), 6.57 (s, 1H, OH), 6.64–6.67 (d, *J*= 8.1 HZ, 1H, –C_4_H), 6.91–6.96 (t, *J*=7.5 Hz, 1H, –C_5_H), 7.11–7.34 (m, 5H, –C_6_H, –C_7_H and Ar—H), 7.45–7.47 (d, *J*=7.2 Hz, 2H, Ar—H),7.56 (bs, 2H, NH_2_); ^13^C NMR (DMSO-*d_6_*): δ 32.67, 42.89, 69.52, 76.02, 108.99, 121.89, 123.72, 126.99, 127.14 (2 C) 127.43, 128.22 (2 C), 129.34, 136.01, 143.15, 171.96 (C=N), 174.42 (isatin C=O), 182.26 (creatinine C=O).

### Refinement

H atoms were found in difference Fourier maps and subsequently placed in idealized positions with constrained distances of 0.98 Å (RCH_3_), 0.99 Å (*R*_2_CH_2_), 1.00 Å (*R*_3_CH), 0.95 Å (C_Ar_H), 0.84 Å (O—H), 0.88 Å (N—H), and with *U*_iso_(H) values set to either 1.2*U*_eq_ or 1.5*U*_eq_ (RCH_3_, OH) of the attached atom.

The benzyl ring is disordered over two positions with refined occupancy factors of 0.847 (7) and 0.153 (7). To ensure stable refinement of the minor component, a number of constraints and restraints were applied. The constraint (an *SHELXL97* EADP instruction on atoms C16 and C16') forces the displacement parameters for these nearly superimposed atoms to be equal. The restraints (*SHELXL97* commands SAME, FLAT, DELU and SIMU) ensure chemically and physically reasonable parameters for the disordered atoms.

The solvent used to grow the crystal was a mixture of ethanol and methanol, but it likely also contained an unknown amount of ethyl acetate. The resulting electron density was largely uninterpretable. It was decided to remove it with the SQUEEZE routine in *PLATON* (Spek, 2009).

### Crystal data

**Table:**

C_19_H_18_N_4_O_3_	*F*(000) = 1472
*M**_r_* = 350.37	*D*_x_ = 1.190 Mg m^−^^3^
Orthorhombic, *P**b**c**a*	Cu *K*α radiation, λ = 1.54178 Å
Hall symbol: -P 2ac 2ab	Cell parameters from 9086 reflections
*a* = 13.4466 (2) Å	θ = 4.6–68.9°
*b* = 10.6921 (2) Å	µ = 0.68 mm^−^^1^
*c* = 27.2057 (5) Å	*T* = 90 K
*V* = 3911.43 (12) Å^3^	Plate, pale yellow
*Z* = 8	0.12 × 0.10 × 0.04 mm

### Data collection

**Table:**

Bruker X8 Proteum CCD diffractometer	3602 independent reflections
Radiation source: fine-focus rotating anode	3344 reflections with *I* > 2σ(*I*)
Graded multilayer optics monochromator	*R*_int_ = 0.043
Detector resolution: 5.6 pixels mm^-1^	θ_max_ = 68.7°, θ_min_ = 4.6°
φ and ω scans	*h* = −16→16
Absorption correction: multi-scan (*SADABS*; Bruker, 2006)	*k* = −12→12
*T*_min_ = 0.911, *T*_max_ = 0.973	*l* = −32→31
55165 measured reflections	

### Refinement

**Table:**

Refinement on *F*^2^	Secondary atom site location: difference Fourier map
Least-squares matrix: full	Hydrogen site location: inferred from neighbouring sites
*R*[*F*^2^ > 2σ(*F*^2^)] = 0.038	H-atom parameters constrained
*wR*(*F*^2^) = 0.104	*w* = 1/[σ^2^(*F*_o_^2^) + (0.0555*P*)^2^ + 1.5834*P*] where *P* = (*F*_o_^2^ + 2*F*_c_^2^)/3
*S* = 1.04	(Δ/σ)_max_ = 0.001
3602 reflections	Δρ_max_ = 0.28 e Å^−^^3^
287 parameters	Δρ_min_ = −0.30 e Å^−^^3^
222 restraints	Extinction correction: *SHELXL97* (Sheldrick, 2008), Fc^*^=kFc[1+0.001xFc^2^λ^3^/sin(2θ)]^-1/4^
Primary atom site location: structure-invariant direct methods	Extinction coefficient: 0.00043 (9)

### Special details

**Table:**

Geometry. All e.s.d.'s (except the e.s.d. in the dihedral angle between two l.s. planes) are estimated using the full covariance matrix. The cell e.s.d.'s are taken into account individually in the estimation of e.s.d.'s in distances, angles and torsion angles; correlations between e.s.d.'s in cell parameters are only used when they are defined by crystal symmetry. An approximate (isotropic) treatment of cell e.s.d.'s is used for estimating e.s.d.'s involving l.s. planes.
Refinement. Refinement of *F*^2^ against ALL reflections. The weighted *R*-factor *wR* and goodness of fit *S* are based on *F*^2^, conventional *R*-factors *R* are based on *F*, with *F* set to zero for negative *F*^2^. The threshold expression of *F*^2^ > 2σ(*F*^2^) is used only for calculating *R*-factors(gt) *etc*. and is not relevant to the choice of reflections for refinement. *R*-factors based on *F*^2^ are statistically about twice as large as those based on *F*, and *R*- factors based on ALL data will be even larger.

### Fractional atomic coordinates and isotropic or equivalent isotropic displacement parameters (Å^2^)

**Table:**

	*x*	*y*	*z*	*U*_iso_*/*U*_eq_	Occ. (<1)
O1	0.34991 (6)	0.33786 (8)	0.64888 (3)	0.0243 (2)	
C1	0.41054 (9)	0.42315 (12)	0.65145 (4)	0.0212 (3)	
N2	0.40105 (8)	0.53751 (10)	0.63017 (4)	0.0240 (2)	
C3	0.48704 (9)	0.61071 (13)	0.63800 (4)	0.0252 (3)	
C4	0.50442 (11)	0.73157 (14)	0.62246 (5)	0.0339 (3)	
H4	0.4551	0.7781	0.6054	0.041*	
C5	0.59772 (12)	0.78247 (14)	0.63292 (6)	0.0396 (4)	
H5	0.6120	0.8657	0.6229	0.047*	
C6	0.66974 (11)	0.71461 (14)	0.65753 (5)	0.0359 (3)	
H6	0.7331	0.7508	0.6635	0.043*	
C7	0.64986 (10)	0.59308 (13)	0.67374 (5)	0.0283 (3)	
H7	0.6990	0.5464	0.6909	0.034*	
C8	0.55730 (9)	0.54224 (12)	0.66432 (4)	0.0231 (3)	
O9	0.56962 (6)	0.31592 (8)	0.66485 (3)	0.0238 (2)	
H9	0.5498	0.2516	0.6796	0.036*	
C9	0.51144 (9)	0.41884 (11)	0.67907 (4)	0.0202 (3)	
C10	0.49128 (8)	0.41474 (11)	0.73572 (4)	0.0180 (3)	
H10	0.4592	0.3339	0.7453	0.022*	
O11	0.34262 (6)	0.54407 (8)	0.74190 (3)	0.0234 (2)	
C11	0.42968 (9)	0.52621 (11)	0.75378 (4)	0.0188 (3)	
N12	0.48658 (7)	0.60115 (9)	0.78275 (4)	0.0200 (2)	
N13	0.65376 (7)	0.59593 (10)	0.80798 (4)	0.0228 (2)	
H13A	0.7113	0.5567	0.8086	0.027*	
H13B	0.6474	0.6691	0.8224	0.027*	
C13	0.57697 (9)	0.54496 (11)	0.78532 (4)	0.0188 (3)	
N14	0.58264 (7)	0.43382 (9)	0.76280 (3)	0.0185 (2)	
C14	0.66413 (9)	0.34513 (12)	0.76781 (5)	0.0240 (3)	
H14A	0.7141	0.3616	0.7424	0.036*	
H14B	0.6386	0.2598	0.7640	0.036*	
H14C	0.6945	0.3543	0.8004	0.036*	
C15	0.31240 (10)	0.58041 (13)	0.60432 (5)	0.0262 (3)	
H15A	0.2929	0.6630	0.6176	0.031*	
H15B	0.2574	0.5214	0.6111	0.031*	
C16	0.3251 (2)	0.5920 (3)	0.54852 (14)	0.0295 (5)	0.847 (7)
C17	0.2898 (2)	0.6974 (3)	0.52528 (8)	0.0565 (8)	0.847 (7)
H17	0.2631	0.7645	0.5440	0.068*	0.847 (7)
C18	0.2932 (3)	0.7056 (3)	0.47406 (8)	0.0724 (11)	0.847 (7)
H18	0.2687	0.7783	0.4580	0.087*	0.847 (7)
C19	0.3315 (2)	0.6099 (3)	0.44696 (10)	0.0573 (8)	0.847 (7)
H19	0.3337	0.6159	0.4121	0.069*	0.847 (7)
C20	0.3668 (3)	0.5051 (4)	0.46988 (15)	0.0397 (7)	0.847 (7)
H20	0.3936	0.4384	0.4509	0.048*	0.847 (7)
C21	0.3636 (3)	0.4957 (4)	0.52102 (15)	0.0313 (7)	0.847 (7)
H21	0.3881	0.4227	0.5368	0.038*	0.847 (7)
C16'	0.3153 (14)	0.5782 (19)	0.5524 (9)	0.0295 (5)	0.153 (7)
C17'	0.2496 (12)	0.6474 (16)	0.5261 (4)	0.054 (2)	0.153 (7)
H17'	0.2047	0.7002	0.5433	0.065*	0.153 (7)
C18'	0.2450 (13)	0.6442 (17)	0.4741 (4)	0.060 (2)	0.153 (7)
H18'	0.1982	0.6935	0.4565	0.072*	0.153 (7)
C19'	0.3107 (13)	0.5673 (16)	0.4500 (6)	0.054 (2)	0.153 (7)
H19'	0.3098	0.5632	0.4151	0.065*	0.153 (7)
C20'	0.3770 (18)	0.497 (2)	0.4756 (9)	0.042 (2)	0.153 (7)
H20'	0.4220	0.4438	0.4587	0.050*	0.153 (7)
C21'	0.3787 (17)	0.503 (2)	0.5264 (9)	0.033 (2)	0.153 (7)
H21'	0.4255	0.4533	0.5439	0.040*	0.153 (7)

### Atomic displacement parameters (Å^2^)

**Table:**

	*U*^11^	*U*^22^	*U*^33^	*U*^12^	*U*^13^	*U*^23^
O1	0.0232 (4)	0.0268 (5)	0.0229 (4)	−0.0029 (4)	−0.0027 (3)	0.0017 (4)
C1	0.0227 (6)	0.0254 (6)	0.0154 (5)	−0.0006 (5)	0.0014 (4)	0.0013 (5)
N2	0.0241 (5)	0.0277 (6)	0.0202 (5)	−0.0014 (4)	−0.0022 (4)	0.0059 (4)
C3	0.0273 (6)	0.0290 (7)	0.0193 (6)	−0.0040 (5)	0.0020 (5)	0.0038 (5)
C4	0.0402 (8)	0.0320 (7)	0.0295 (7)	−0.0029 (6)	−0.0008 (6)	0.0104 (6)
C5	0.0493 (9)	0.0330 (8)	0.0364 (8)	−0.0140 (7)	0.0008 (7)	0.0116 (6)
C6	0.0373 (8)	0.0409 (8)	0.0295 (7)	−0.0162 (6)	0.0000 (6)	0.0063 (6)
C7	0.0280 (7)	0.0359 (7)	0.0210 (6)	−0.0067 (6)	0.0015 (5)	0.0040 (5)
C8	0.0257 (6)	0.0276 (6)	0.0161 (6)	−0.0036 (5)	0.0023 (5)	0.0027 (5)
O9	0.0236 (4)	0.0257 (5)	0.0221 (4)	0.0010 (3)	0.0040 (3)	−0.0004 (3)
C9	0.0199 (6)	0.0230 (6)	0.0178 (6)	−0.0008 (5)	0.0006 (4)	0.0010 (4)
C10	0.0172 (6)	0.0189 (6)	0.0178 (6)	−0.0008 (4)	−0.0004 (4)	0.0016 (4)
O11	0.0178 (4)	0.0229 (4)	0.0295 (5)	0.0006 (3)	−0.0024 (3)	0.0009 (3)
C11	0.0181 (6)	0.0203 (6)	0.0179 (5)	−0.0011 (4)	0.0011 (4)	0.0040 (4)
N12	0.0182 (5)	0.0209 (5)	0.0208 (5)	0.0006 (4)	−0.0003 (4)	−0.0001 (4)
N13	0.0191 (5)	0.0234 (5)	0.0258 (5)	0.0024 (4)	−0.0041 (4)	−0.0046 (4)
C13	0.0196 (6)	0.0223 (6)	0.0146 (5)	−0.0002 (5)	0.0007 (4)	0.0031 (4)
N14	0.0181 (5)	0.0187 (5)	0.0186 (5)	0.0014 (4)	−0.0015 (4)	0.0001 (4)
C14	0.0198 (6)	0.0215 (6)	0.0308 (6)	0.0028 (5)	−0.0044 (5)	−0.0001 (5)
C15	0.0248 (6)	0.0340 (7)	0.0199 (6)	0.0057 (5)	0.0006 (5)	0.0044 (5)
C16	0.0267 (10)	0.0427 (11)	0.0191 (9)	0.0057 (7)	0.0021 (7)	0.0076 (7)
C17	0.0808 (19)	0.0593 (16)	0.0294 (9)	0.0366 (14)	0.0088 (11)	0.0144 (10)
C18	0.106 (2)	0.078 (2)	0.0330 (10)	0.0440 (19)	0.0066 (13)	0.0227 (12)
C19	0.0693 (17)	0.083 (2)	0.0196 (9)	0.0199 (15)	0.0079 (10)	0.0136 (12)
C20	0.0358 (13)	0.0602 (14)	0.0230 (14)	0.0012 (11)	0.0021 (10)	−0.0050 (11)
C21	0.0316 (15)	0.0380 (11)	0.0243 (14)	−0.0012 (10)	−0.0027 (9)	−0.0008 (9)
C16'	0.0267 (10)	0.0427 (11)	0.0191 (9)	0.0057 (7)	0.0021 (7)	0.0076 (7)
C17'	0.068 (4)	0.064 (4)	0.030 (3)	0.024 (4)	0.005 (4)	0.012 (4)
C18'	0.081 (5)	0.072 (5)	0.026 (3)	0.031 (4)	−0.002 (4)	0.019 (4)
C19'	0.069 (4)	0.075 (5)	0.019 (4)	0.014 (4)	−0.001 (4)	0.008 (4)
C20'	0.044 (4)	0.059 (4)	0.023 (4)	0.005 (4)	0.000 (4)	0.001 (4)
C21'	0.029 (4)	0.051 (4)	0.020 (4)	−0.003 (4)	−0.001 (4)	0.004 (4)

### Geometric parameters (Å, º)

**Table:**

O1—C1	1.2253 (15)	C14—H14A	0.9800
C1—N2	1.3588 (16)	C14—H14B	0.9800
C1—C9	1.5516 (16)	C14—H14C	0.9800
N2—C3	1.4124 (17)	C15—C16'	1.41 (2)
N2—C15	1.4582 (16)	C15—C16	1.533 (4)
C3—C4	1.3796 (19)	C15—H15A	0.9900
C3—C8	1.3933 (18)	C15—H15B	0.9900
C4—C5	1.397 (2)	C16—C21	1.374 (3)
C4—H4	0.9500	C16—C17	1.377 (3)
C5—C6	1.383 (2)	C17—C18	1.397 (3)
C5—H5	0.9500	C17—H17	0.9500
C6—C7	1.398 (2)	C18—C19	1.362 (4)
C6—H6	0.9500	C18—H18	0.9500
C7—C8	1.3821 (18)	C19—C20	1.368 (4)
C7—H7	0.9500	C19—H19	0.9500
C8—C9	1.5107 (17)	C20—C21	1.396 (3)
O9—C9	1.4046 (15)	C20—H20	0.9500
O9—H9	0.8400	C21—H21	0.9500
C9—C10	1.5654 (16)	C16'—C17'	1.357 (16)
C10—N14	1.4468 (14)	C16'—C21'	1.368 (17)
C10—C11	1.5322 (16)	C17'—C18'	1.418 (13)
C10—H10	1.0000	C17'—H17'	0.9500
O11—C11	1.2295 (14)	C18'—C19'	1.373 (15)
C11—N12	1.3597 (16)	C18'—H18'	0.9500
N12—C13	1.3576 (15)	C19'—C20'	1.359 (16)
N13—C13	1.3204 (15)	C19'—H19'	0.9500
N13—H13A	0.8800	C20'—C21'	1.385 (17)
N13—H13B	0.8800	C20'—H20'	0.9500
C13—N14	1.3392 (16)	C21'—H21'	0.9500
N14—C14	1.4555 (15)		
			
O1—C1—N2	125.66 (11)	N14—C14—H14B	109.5
O1—C1—C9	125.97 (11)	H14A—C14—H14B	109.5
N2—C1—C9	108.37 (10)	N14—C14—H14C	109.5
C1—N2—C3	110.95 (10)	H14A—C14—H14C	109.5
C1—N2—C15	124.43 (11)	H14B—C14—H14C	109.5
C3—N2—C15	124.59 (11)	C16'—C15—N2	117.0 (8)
C4—C3—C8	122.34 (12)	N2—C15—C16	114.35 (15)
C4—C3—N2	127.69 (12)	C16'—C15—H15A	112.9
C8—C3—N2	109.96 (11)	N2—C15—H15A	108.7
C3—C4—C5	117.03 (13)	C16—C15—H15A	108.7
C3—C4—H4	121.5	C16'—C15—H15B	101.4
C5—C4—H4	121.5	N2—C15—H15B	108.7
C6—C5—C4	121.55 (13)	C16—C15—H15B	108.7
C6—C5—H5	119.2	H15A—C15—H15B	107.6
C4—C5—H5	119.2	C21—C16—C17	119.6 (3)
C5—C6—C7	120.43 (13)	C21—C16—C15	121.4 (3)
C5—C6—H6	119.8	C17—C16—C15	118.9 (3)
C7—C6—H6	119.8	C16—C17—C18	119.8 (3)
C8—C7—C6	118.64 (13)	C16—C17—H17	120.1
C8—C7—H7	120.7	C18—C17—H17	120.1
C6—C7—H7	120.7	C19—C18—C17	120.4 (2)
C7—C8—C3	119.95 (12)	C19—C18—H18	119.8
C7—C8—C9	131.44 (12)	C17—C18—H18	119.8
C3—C8—C9	108.58 (11)	C18—C19—C20	120.0 (3)
C9—O9—H9	109.5	C18—C19—H19	120.0
O9—C9—C8	112.56 (10)	C20—C19—H19	120.0
O9—C9—C1	112.14 (10)	C19—C20—C21	120.2 (3)
C8—C9—C1	101.67 (9)	C19—C20—H20	119.9
O9—C9—C10	110.23 (9)	C21—C20—H20	119.9
C8—C9—C10	110.90 (9)	C16—C21—C20	120.0 (3)
C1—C9—C10	109.04 (9)	C16—C21—H21	120.0
N14—C10—C11	100.73 (9)	C20—C21—H21	120.0
N14—C10—C9	110.50 (9)	C17'—C16'—C21'	117.0 (18)
C11—C10—C9	112.80 (9)	C17'—C16'—C15	120.0 (18)
N14—C10—H10	110.8	C21'—C16'—C15	123.0 (16)
C11—C10—H10	110.8	C16'—C17'—C18'	122.8 (14)
C9—C10—H10	110.8	C16'—C17'—H17'	118.6
O11—C11—N12	126.63 (11)	C18'—C17'—H17'	118.6
O11—C11—C10	123.45 (11)	C19'—C18'—C17'	117.6 (12)
N12—C11—C10	109.88 (10)	C19'—C18'—H18'	121.2
C13—N12—C11	105.83 (10)	C17'—C18'—H18'	121.2
C13—N13—H13A	120.0	C20'—C19'—C18'	120.5 (16)
C13—N13—H13B	120.0	C20'—C19'—H19'	119.8
H13A—N13—H13B	120.0	C18'—C19'—H19'	119.8
N13—C13—N14	122.37 (11)	C19'—C20'—C21'	119.9 (19)
N13—C13—N12	122.79 (11)	C19'—C20'—H20'	120.0
N14—C13—N12	114.84 (10)	C21'—C20'—H20'	120.0
C13—N14—C10	108.04 (9)	C16'—C21'—C20'	122.2 (19)
C13—N14—C14	125.31 (10)	C16'—C21'—H21'	118.9
C10—N14—C14	126.52 (10)	C20'—C21'—H21'	118.9
N14—C14—H14A	109.5		
			
O1—C1—N2—C3	−176.47 (11)	O11—C11—N12—C13	−179.47 (11)
C9—C1—N2—C3	3.04 (13)	C10—C11—N12—C13	−1.45 (12)
O1—C1—N2—C15	5.67 (19)	C11—N12—C13—N13	175.47 (11)
C9—C1—N2—C15	−174.83 (10)	C11—N12—C13—N14	−4.15 (13)
C1—N2—C3—C4	−179.33 (13)	N13—C13—N14—C10	−171.44 (10)
C15—N2—C3—C4	−1.5 (2)	N12—C13—N14—C10	8.18 (13)
C1—N2—C3—C8	1.47 (14)	N13—C13—N14—C14	12.39 (18)
C15—N2—C3—C8	179.33 (11)	N12—C13—N14—C14	−167.99 (10)
C8—C3—C4—C5	1.9 (2)	C11—C10—N14—C13	−7.92 (11)
N2—C3—C4—C5	−177.19 (13)	C9—C10—N14—C13	111.54 (10)
C3—C4—C5—C6	0.3 (2)	C11—C10—N14—C14	168.19 (10)
C4—C5—C6—C7	−1.5 (2)	C9—C10—N14—C14	−72.35 (14)
C5—C6—C7—C8	0.5 (2)	C1—N2—C15—C16'	−101.9 (9)
C6—C7—C8—C3	1.71 (19)	C3—N2—C15—C16'	80.6 (9)
C6—C7—C8—C9	−176.14 (13)	C1—N2—C15—C16	−109.52 (19)
C4—C3—C8—C7	−3.0 (2)	C3—N2—C15—C16	72.9 (2)
N2—C3—C8—C7	176.26 (11)	C16'—C15—C16—C21	−62 (8)
C4—C3—C8—C9	175.30 (12)	N2—C15—C16—C21	50.4 (3)
N2—C3—C8—C9	−5.45 (14)	C16'—C15—C16—C17	113 (8)
C7—C8—C9—O9	−55.07 (17)	N2—C15—C16—C17	−134.8 (2)
C3—C8—C9—O9	126.90 (11)	C21—C16—C17—C18	0.11 (19)
C7—C8—C9—C1	−175.25 (13)	C15—C16—C17—C18	−174.8 (2)
C3—C8—C9—C1	6.72 (12)	C16—C17—C18—C19	−0.1 (2)
C7—C8—C9—C10	68.94 (17)	C17—C18—C19—C20	0.0 (4)
C3—C8—C9—C10	−109.09 (11)	C18—C19—C20—C21	0.1 (4)
O1—C1—C9—O9	53.15 (15)	C17—C16—C21—C20	0.0 (3)
N2—C1—C9—O9	−126.36 (11)	C15—C16—C21—C20	174.8 (2)
O1—C1—C9—C8	173.61 (11)	C19—C20—C21—C16	−0.1 (4)
N2—C1—C9—C8	−5.89 (12)	N2—C15—C16'—C17'	−161.4 (9)
O1—C1—C9—C10	−69.22 (15)	C16—C15—C16'—C17'	−91 (8)
N2—C1—C9—C10	111.28 (11)	N2—C15—C16'—C21'	22.3 (12)
O9—C9—C10—N14	67.00 (12)	C16—C15—C16'—C21'	93 (8)
C8—C9—C10—N14	−58.33 (12)	C21'—C16'—C17'—C18'	−0.1 (3)
C1—C9—C10—N14	−169.48 (10)	C15—C16'—C17'—C18'	−176.5 (14)
O9—C9—C10—C11	178.88 (9)	C16'—C17'—C18'—C19'	−0.1 (3)
C8—C9—C10—C11	53.55 (13)	C17'—C18'—C19'—C20'	0.2 (7)
C1—C9—C10—C11	−57.60 (12)	C18'—C19'—C20'—C21'	−0.2 (9)
N14—C10—C11—O11	−176.13 (11)	C17'—C16'—C21'—C20'	0.1 (7)
C9—C10—C11—O11	66.08 (14)	C15—C16'—C21'—C20'	176.5 (14)
N14—C10—C11—N12	5.78 (12)	C19'—C20'—C21'—C16'	0.0 (10)
C9—C10—C11—N12	−112.01 (11)		

### Hydrogen-bond geometry (Å, º)

**Table:**

*D*—H···*A*	*D*—H	H···*A*	*D*···*A*	*D*—H···*A*
O9—H9···N12^i^	0.84	1.97	2.8065 (13)	175
N13—H13*A*···O11^ii^	0.88	2.24	2.9321 (13)	135
N13—H13*B*···O1^iii^	0.88	1.97	2.8410 (14)	173

Symmetry codes: (i) −*x*+1, *y*−1/2, −*z*+3/2; (ii) *x*+1/2, *y*, −*z*+3/2; (iii) −*x*+1, *y*+1/2, −*z*+3/2.
